# Fatal course of idiopathic chronic ulcerative enteritis with panenteritis and perforation: a case report and review of literature

**DOI:** 10.1186/s12893-020-00850-4

**Published:** 2020-09-07

**Authors:** Chang-Yeon Jung, Jung-Min Bae

**Affiliations:** Department of Surgery, Yeungnam University Medical Center, 170 Hyeonchung-ro, Nam-gu, Daegu, 42415 Republic of Korea

**Keywords:** Enteritis, Intestine, Ulcer

## Abstract

**Background:**

Idiopathic chronic ulcerative enteritis (ICUE) is a very rare disease with high mortality. Because of clinical rarity, several small case reports have been published and there is a lack of large sample study. Preoperative definite diagnosis is difficult. Although definite treatment for ICUE is radical surgical resection, surgical decision in operative field is difficult.

**Case presentation:**

A 77-year-old man came to the emergency department with complaints of a 1-day history of abdominal pain and abdominal distension. Abdominal computed tomography revealed ileus and focal free air. Laparotomy revealed multiple small bowel tiny perforations in the ileum. The serosa surface in the whole small bowel had small multiple yellowish tiny discolored lesions. Despite the presence of multiple mucosal ulcers in entire small bowel, the ileum including perforation site was resected segmentally. Microscopically, mucosal ulcers in resected small bowel demonstrated transmural inflammation, no granuloma, and no lymphoid aggregates. These features were consistent with a diagnosis of ICUE with panenteritis and perforation. After surgery, the patient’s general condition gradually aggravated. Unfortunately, the patient died of multiple organ failure on post-operative day 14.

**Conclusion:**

Surgically, the decision including resection range, anastomosis or enterotomy becomes difficult in ICUE with panenteritis. According to recent 40 year’s revised data, the post-operative mortality of ICUE is about 53.4%. Although ICUE is rare, its recognition is important for appropriate diagnosis and treatment. Retrospective multicenter case studies are required to determine proper treatment and improve prognosis.

## Background

Idiopathic chronic ulcerative enteritis (ICUE) is a rare disease and diagnosis of ICUE is very difficult before laparotomy. The etiology of ICUE remains unknown.

Historically, multiple diffuse nonspecific nongranulomatous small intestinal ulcer without underlying disease was first described in 1949 as a kind of ulcerous jejuno-ileitis [1].

Additionally, ICUE is defined in terms of several different descriptive phrases including ulcerative jejunitis, ulcerative jejunoileitis, nongranulomatous ulcerative jejunoileitis, ulcerative enteritis, and nonspecific ulcerative duodenojejunoileitis [2, 3].

ICUE patients have chronic gastrointestinal symptoms. However, most of the ICUE cases remained undiagnosed until laparotomy. Generally, laparotomy is performed in emergency settings because of intestinal obstruction, perforation, and bleeding. Finally, after laparotomy, the diagnosis of ICUE becomes feasible in most of the cases of ICUE.

In the current study, we present a case of a 77-year-old male patient diagnosed with ICUE with panenteritis and perforation. Despite surgical treatment, post-operative mortality occured.

Also, we have included a review of the literature.

This study was approved by the Institutional Review Board of Yeungnam University Medical Center (IRB No. 2020–01-037). The patient provided written informed consent regarding the publication of case details at admission.

## Case presentation

A 77-year-old man came to the emergency department with complaints of a 1-day history of abdominal pain and abdominal distension.

His family history about gastrointestinal disorder was non-specific. His past-medical history including non-steroidal anti-inflammatory drug (NSAID) medication was non-specific.

The initial blood pressure was 160/70 mmHg, heart rate was 86/min, respiratory rate was 20/min, and body temperature was 36.9 °C at admission. Initial laboratory findings revealed slight elevation of leucocytes and hemoglobin at 11.9 g/dL.

The bowel sounds were silent. Physical examination revealed tenderness in the periumbilical area with rebound tenderness and guarding.

**Fig. 1:**
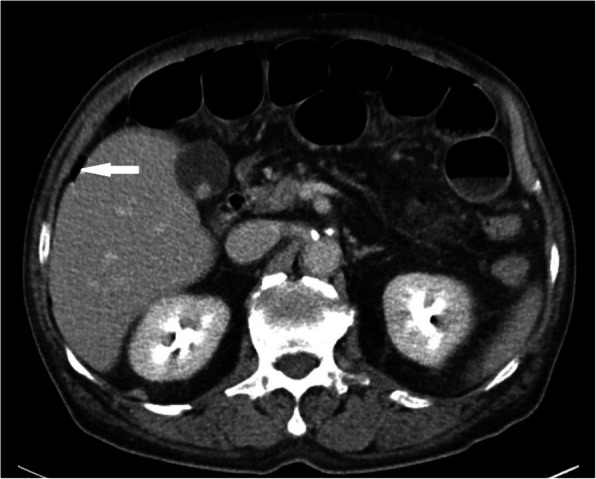
Abdominal computed tomographic findings. Free air in liver surface (white arrow) were observed

Abdominal computed tomography revealed ileus with edematous small bowel loops, mild ascites, non-specific mass lesions, and focal free air in the right upper quadrant (Fig. 1).

The results led us to consider peritonitis due to small bowel perforation. The surgical procedure was indicated and emergency exploration was performed thereafter.

**Fig. 2:**
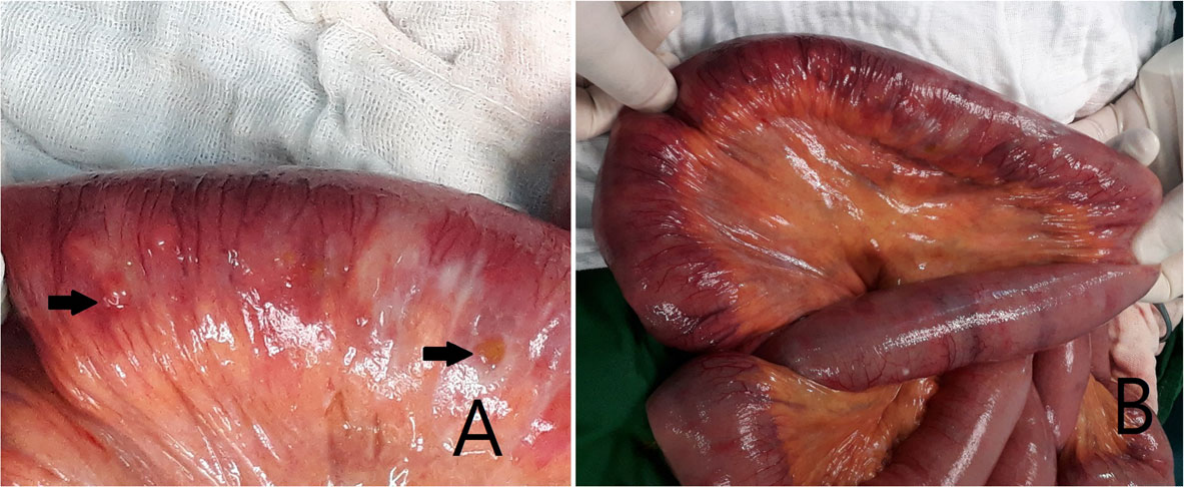
Gross findings of ICUE. **a** The perforation sites were in the form of very small holes in mesenteric border (black arrows). **b** The serosa surface in the whole small bowel had small multiple yellowish tiny discolored lesions near the mesenteric border

Laparotomy revealed multiple small bowel tiny perforations in the ileum. The perforation sites were in the form of very small holes and measured about 0.1 ~ 0.2 cm in size. Interestingly, all the perforation sites were located in the mesenteric border in the small bowel (Fig. 2a).

The serosa surface in the entire small bowel had small multiple yellowish tiny discolored lesions near the mesenteric border in the small bowel (Fig. 2b).

These discolored lesions in the resected bowel and both proximal and distal remnant bowel were equal to luminal ulcer locations in the inner surface. Serosal discolored tiny oval-shaped lesions from Treitz ligament to ileocecal valve were considered as luminal ulcers.

About 78 cm of the ileum including perforation site was resected.

However, remnant small bowel had multiple small ulcers in the mesenteric border.

**Fig. 3:**
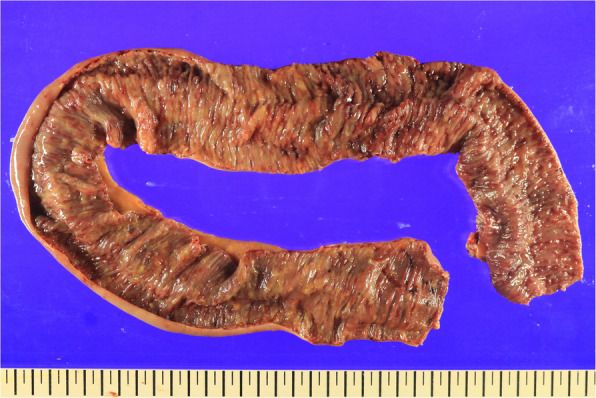
The longitudinally cut surface of the resected small bowel revealed the presence of dirty, friable mucosa, and multiple tiny ulcers

The longitudinally cut surface of the resected small bowel revealed the presence of friable mucosa, and multiple tiny ulcers (Fig. 3).

Despite the presence of multiple mucosal ulcers in small bowel including proximal and distal small bowel, we should aim to preserve small bowel with ulcer considering postoperative short bowel syndrome, old age, and poor general status. Primary end-to end stapled anastomosis of proximal and distal small bowel was performed.

Microscopically, mucosal ulcers in resected small bowel demonstrated transmural inflammation, no granuloma, and no lymphoid aggregates.. Fibrosis or fat wrapping was not seen. The ulcers were oval-shaped with varying depths. Deep ulcers reached the serosa and formed transmural perforation without granuloma.

The grossly small bowel ulcers and histologic features were consistent with a diagnosis of ICUE with panenteritis and perforation.

After surgery, the patient’s course was uneventful until postoperative day 4. However, on day 5, intermittent hematochezia, melena, and greenish diarrhea developed repeatedly without hemodynamic instability. Blood transfusion, fluid infusion, and intravenous administration of coagulant were performed.

Despite these conservative treatments, the patient’s general condition gradually aggravated due to recurrent hematochezia and poor nutritional status. Unfortunately, the patient died of multiple organ failure on post-operative day 14.

## Discussion and conclusions

Idiopathic chronic ulcerative enteritis (ICUE) is defined as multiple diffuse nonspecific nongranulomatous small intestinal ulcerative disease with unknown etiology.

Most of the ICUE patients in previously reported literature had chronic gastrointestinal symptoms.

In the early stage of ICUE, the symptom is ambiguous. However, general presentations in ICUE are weight loss, chronic abdominal pain, diarrhea, melena, hematochezia, a positive fecal occult blood test, malnutrition, iron deficiency anemia, hypoalbuminemia, and so on [4, 5].

However, clinical features vary including the asymptomatic case, chronic gastro-intestinal symptoms, and acute abdominal pain. Although these symptoms are helpful for suspicion of small bowel disease, they are of no aid for early diagnosis of ICUE. In some cases without a history of chronic symptoms, diagnosis of ICUE has been made [5, 6].

Under conditions of the above-mentioned presentations, several diagnostic examinations are recommended.

About 40 years back, per-oral jejunal biopsy and small bowel series were performed for the diagnosis of small bowel abnormality [6].

However, with recent developments of radiologic tests and endoscopic equipment, gastro-endoscopy, colonoscopy, double-balloon enteroscopy, computed tomography, and capsule endoscopy are considered for the diagnosis [2, 7]. In Korea, 4650 patients with obscure gastrointestinal bleeding, chronic abdominal pain, and weight loss similar to ICUE symptoms were evaluated by capsule endoscopy. Among these, small bowel ulcer was revealed in 10% of patients [7].

However, these radiologic and endoscopic examinations are not effective in the definite diagnosis of ICUE. Most of the ICUE cases remain undiagnosed until laparotomy.

Finally, the definite diagnosis of ICUE is made by pathological evaluation of resected small bowel specimens after laparotomy.

When a patient has small bowel obstruction, perforation, bleeding, chronic malnutrition or weight loss related gastrointestinal function, laparotomy is performed [2, 4, 6, 8–10].

Among 43 patients, bowel perforation developed in 14 patients and bowel obstruction developed in 19 patients (Table 1) [6].

**Table 1:** Clinical and prognostic data of 43 cases of ICUE

	N	Age	Sex	Chronic Hx duration	Cause of Operation	Post-operative outcome
Mills PR (1980) [6]	32	–	–	–	Perforation: 9	survival: 12
					Obstruction: 9	death: 20
Lamont C.M. (1982) [10]		62	F	2 yr	small bowel obstruction	survival
		63	F	4 m	small bowel obstruction	survival
Hinder R.A (1985) [9]		54	m	6	jejunal obstruction	survival
Zapolsky J.H. (1985) [5]		65	m	*	small bowel obstruction	survival
		44	m	10 year	jejunal partial obstruction	survival
Breen E.G. (1989) [8]		40	m	2	duodenal 4th portion obstruction	survival
Sutton C.D. (2002) [4]		65	m	*	small bowel perforation	death
		38	m	*	jejunal perforation	survival
		49	m	*	jejunal perforation	survival
Gao X. (2013) [2]		78	m	4	small Bowel perforation	death
Our case		77	m	*	ileal perforation	death

In the operative view on laparotomy, several characteristic features of ICUE were revealed.

The common findings include bowel thickening, edema, and serosal hyperemia. Mesenteric lymphadenopathy was often seen [6].

Small bowel ulcers are more common in the jejunum than ileum and duodenal or colonic involvement of ulcers is rare. However, the severity of ulcer or involved range in small bowel was not described in detail [6].

Surgically, if there is diffuse involvement of small bowel, the decision about the resection range becomes difficult. Though small bowel with perforation or bleeding should be resected, wide resection of diffuse small bowel ulceration is difficult because surplus small bowel resection causes short bowel syndrome.

In this panenteritis condition, wide resection of small bowel should be performed considering the prevention of post-operative short bowel syndrome. However, recurrent bleeding, perforation, and obstruction may develop in remnant small bowel ulcers. Therefore, in the natural disease course of ICUE, several repeated laparotomy might occasionally be needed [6].

Additionally, after small bowel resection, there exists considerable operative maneuver selection between bowel anastomosis and double-lumen enterostomy for circumvention of anastomotic leakage. The edematous small bowel and poor nutritional status could have led to anastomotic leakage [2]. On the contrary, enterostomy of too short proximal remnant small bowel induces short bowel syndrome. In present case, despite edematous remnant small bowel, we selected adequate part for safe anastomosis. In post-operative period, anastomotic leakage was not happened.

Grossly and histopathologically, resected small bowel in ICUE showed multiple small mucosal ulcers with a varying depth between superficial and nearly transmural perforation [2, 3, 10].

The ulcers exhibited round or oval appearance with a diameter ranging from 0.3 ~ 0.5 cm and were located in the mesenteric margin [2]. Microscopic features of mesenteric lymphadenopathy revealed non-specific findings [6]. The granuloma seen in Crohn’s disease was absent [4, 10]. The non-ulcerated mucosa may show villous atrophy [6].

Additionally, we need to differentiate ICUE from small bowel ulcerative disease including Behcet’s disease, NSAID-induced enteropathy, cryptogenic multifocal ulcerous enteritis (CMUSE), and chronic nonspecific multiple ulcers (CNSU).

Behcet’s disease is characterized by the presence of ileocecal ulcer along with transmural inflammation and crater-shaped ulcer margins. Also, Behcet’s disease has a triad of symptoms consisting of aphthous stomatitis, genital ulcers, and ocular symptoms [2]. However, the present case did not exhibit any of the above mentioned clinical characteristics.

NSAID-induced enteropathy has a characteristic narrow ileal stenosis that is called as ‘diaphragm disease’ [11]. However, our case had no NSAID medication history no ileal stricture.

CMUSE is characterized by the presence of small intestinal stricture and superficial mucosal or submucosal ulcer in the small bowel [7, 12]. However, the present case did not exhibit small bowel stricture and multiple superficial and deep ulcers. Therefore, we excluded the presence of CMUSE.

Chronic nonspecific multiple ulcers (CNSU) are different from ICUE. CNSU is limited to only mucosal and submucosal small intestinal ulcers. However, ICUE has variable ulcer depth ranging from mucosa to transmural depth including perforation [12].

Medical treatment including steroid or gluten-free diet is not effective in ICUE [2, 6].

Definite treatment for ICUE is radical surgical resection. Furthermore, repeated laparotomy is required frequently because of symptom recurrence [6].

Despite surgical treatment, post-operative aggravation of the ulcer may develop like intestinal bleeding or perforation. These aggravations may preclude reoperation and lead to death [2, 4].

For analysis of prognosis in ICUE, we reviewed the clinical data of the previously reported cases of ICUE and our case (Table 1).

The frequently cited post-operative mortality rate of ICUE was 62.5% in the report published 40 years back [6]. However, according to recent 40 year’s revised data, the post-operative mortality of ICUE is about 53.4% with deaths in 23 of 43 reported cases (Table 1) [6].

In conclusion, we present a case of ICUE with panenteritis and perforation treated by wide small bowel resection. The diagnosis of ICUE with panenteritis is very difficult before laparotomy. Surgically, the decision including resection range, anastomosis or enterotomy becomes difficult in ICUE.

Despite definite surgical treatment, postoperative intestinal bleeding developed and the patient died of multiple organ failure. According to recent 40 year’s revised data, the post-operative mortality of ICUE is about 53.4%. Although ICUE is rare, its recognition is important for appropriate diagnosis and treatment. Retrospective multicenter case studies are required to determine proper treatment and improve prognosis.

## Data Availability

Data sharing is not applicable to this article, as no datasets were generated or analyzed during the current study.

## Abbreviations

ICUE: Idiopathic chronic ulcerative enteritis
NSAID: Non-steroidal anti-inflammatory drug
CMUSE: Cryptogenic multifocal ulcerous enteritis
CNSU: Chronic nonspecific multiple ulcers
